# The Influence of Sewage Sludge Content and Sintering Temperature on Selected Properties of Lightweight Expanded Clay Aggregate

**DOI:** 10.3390/ma14123363

**Published:** 2021-06-17

**Authors:** Jolanta Latosińska, Maria Żygadło, Przemysław Czapik

**Affiliations:** 1 Faculty of Environmental Engineering, Geomatic and Energy Engineering, Kielce University of Technology, Al. Tysiąclecia Państwa Polskiego 7, 25-314 Kielce, Poland; zygadlo@tu.kielce.pl; 2 Faculty of Civil Engineering and Architecture, Kielce University of Technology, Al. Tysiąclecia Państwa Polskiego 7, 25-314 Kielce, Poland; p.czapik@tu.kielce.pl

**Keywords:** lightweight expanded clay aggregate, sewage sludge, sintering temperature, density, porosity, water absorption, statistical model, recycling

## Abstract

Wastewater treatment processes produce sewage sludge (SS), which, in line with environmental sustainability principles, can be a valuable source of matter in the production of lightweight expanded clay aggregate (LECA). The literature on the influence of SS content and sintering temperature on the properties of LECA is scarce. This paper aims to statistically evaluate the effects of SS content and sintering temperature on LECA physical properties. Total porosity, pore volume, and apparent density were determined with the use of a density analyzer. A helium pycnometer was utilized to determine the specific density. Closed porosity was calculated. The test results demonstrated a statistically significant influence of the SS content on the specific density and water absorption of LECA. The sintering temperature had a significant effect on the specific density, apparent density, total porosity, closed porosity, total volume of pores, and water absorption. It was proved that a broad range of the SS content is admissible in the raw material mass for the production of LECA.

## 1. Introduction

Sewage sludge is an inevitable by-product of municipal wastewater treatment. The amount of sewage sludge generated in the EU is constantly growing, and prognoses indicate the continuation of this trend [1,2,3]. Though sewage sludge constitutes only 1–2% of the treated wastewater volume, the cost of its treatment and disposal reaches 60% of the total cost of wastewater treatment plant operations [4]. Sewage sludge is used in agriculture for its soil-forming and fertilizing properties. However, due to the risk of passing heavy metals, trace organic pollutants, and pathogenic organism from the sludge into the environment, its application is largely restricted [5,6,7,8,9]. Formerly common landfilling practice has been banned in the European Union [10,11]. The recovery of energy from sewage sludge in power plants and mono-incineration plants generates wastes despite reductions in waste volume and mass and elimination of harmful substances [12]. Waste-free utilization of sewage sludge together with the reduction in natural raw material use is desirable in the context of environmental sustainability. One of the solutions is the use of municipal sewage sludge in the production of LECA.

Light expanded clay aggregate (LECA) is produced from natural raw materials in a rotary kiln by sintering at 1100–1200 °C [13,14]. LECA is commonly used in civil engineering, gardening, geotechnology, and environmental engineering [13,14,15,16,17,18,19]. This wide and varied range of applications is due to its characteristic properties: regular, almost spherical shape of particles, surface glazing, total porosity up to 80%, and water absorption up to 20% [13,14,20]. Moreover, it is fireproof, sound-absorbing, and resistant to chemical factors, mold, fungi, insects, and rodents [13,14,17].

The utilization of wastes in the production of LECA started in the 1990s [21]. Natural raw material was supplemented with waste glauconite [22], waste incineration bottom ash [23], fly ash [24], washings from a granite quarry [25], digested spent bentonite clay or basalt powder [26]. The tests have shown that also municipal sewage sludge containing 40–80% dry mass (d.m.) of the organic matter promotes the formation of the porous structure of LECA [27,28,29]. However, the influence of sewage sludge content and sintering temperature on the properties of LECA has never been fully tested. Yan et al. [23] studied the LECA sintering process, concentrating on the effects of sintering temperature, its rise rate, and duration. They found that the temperature rise rate had the least significant effect on the LECA properties and that sintering time had the most significant effect [23].

The aim of the present experiment was to evaluate the influence of both the sewage sludge content in the range of 0–30% and the sintering temperature in the range of 1100–1200 °C on the physical properties of LECA, such as specific density, apparent density, closed porosity, total porosity, total pore volume, and water absorption ability.

## 2. Materials and Methods

### 2.1. Materials

The clay was collected from the deposit in Budy Mszczonowskie (Mszczonów, Poland), formerly exploited by the LECA production plant. The sewage sludge, stabilized and dewatered, was from the mechanical–biological wastewater treatment plant located in Sitkówka–Nowiny (Sitkówka-Nowiny, Poland). The wastewater treatment plant receives sewage from the Kielce metropolitan area.

### 2.2. Experiment Parameters According to the Rotatable Plan

**Figure 1:**
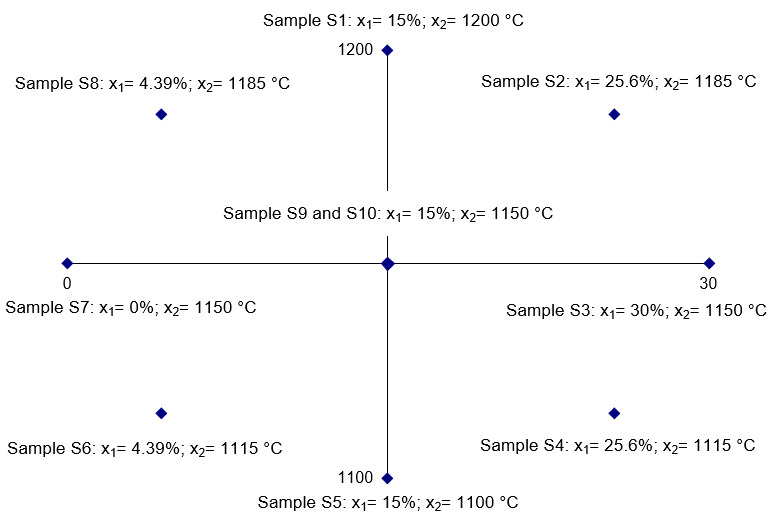
Points determined according to the rotatable plan: x_1_—sewage sludge content % d.m.; x_2_—sintering temperature °C.

In order to increase the effectiveness of the regression function, a rotatable plan was adopted for two independent variables: sewage sludge content (x_1_), and sintering temperature (x_2_; Figure 1). The experiment covered five independent repetitions at every point of the plan.

The sewage sludge content (0–30% d.m.) and the sintering temperature (1100–1200 °C) were established based on the authors’ own former test results [27,28] and the literature data [30,31].

Insignificant coefficients of the multiple regression function were eliminated with the Backward method (the elimination method) for the level of significance *p* < 0.1.

The quadratic model of the multiple regression was used for the adopted rotatable plan [32]:

y_j_ = a_0_ + a_1_ · x_1_ + a_2_ · x_2_ + a_11_ · x_12_ + a_12_ · x_1_ · x_2_ + a_22_ · x_22_ (1)

where x_1_ is sewage sludge content; x_2_ is sintering temperature; a_0_, a_1_, a_2_, a_11_, a_12_, a_12_, and a_22_ are coefficients for independent variables estimated with the least-squares method; and y_j_ represents dependent variables representing specific density, apparent density, total porosity, closed porosity, total volume of pores, and water absorption.

The significance of coefficients in Equation (1) were verified with an *F*-test [32]:

(2) $$F=\frac{N-K-1}{K}\cdot \frac{R^{2}}{1-R^{2}}$$

where R is coefficient of multiple correlation; and N is number of coefficients of the regression function (i.e., a_1_, a_2_, a_11_, a_12_, a_12_, a_22_).

### 2.3. The Conditions of Forming and Sintering Procedures

The raw clay was dried until it reached the air-dry state, ground in a disk-screw grinder, and pulverized in a mortar to fractions <1.0 mm. The sewage sludge was dried at 105 °C until the air-dry state and pulverized in a mortar to fractions <1.0 mm. Raw masses were prepared (150 g) with a variable sewage sludge content (Figure 1).

**Figure 2:**
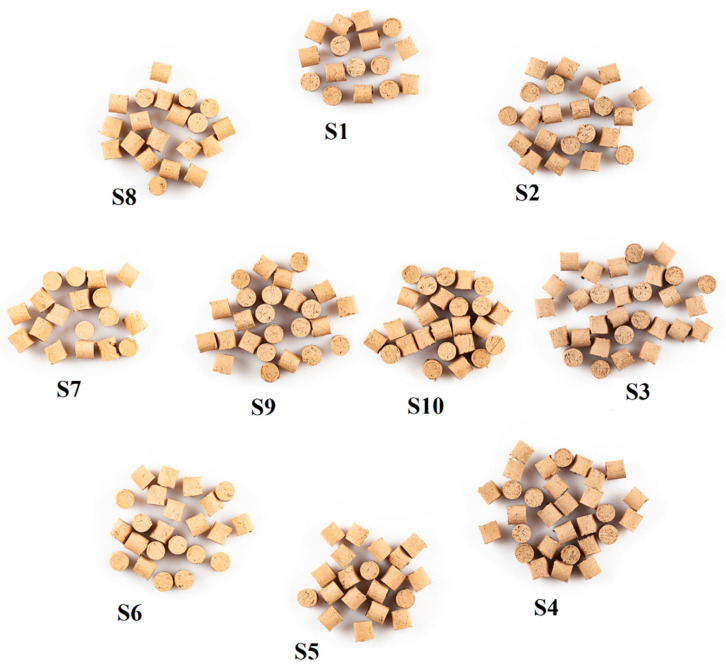
Dried S1–S10 samples.

The sewage sludge and clay were mixed with distilled water until the mixture reached a plastic state. The content of mixing water was 27–31%. After that, cylindrical pieces 10 mm in diameter and 10 mm high (Figure 2) were made with a hand press. The samples were air-dried at room temperature of ~20 °C and then in a laboratory drier (WAMED, Warsaw, Masovian, Poland) at 105 °C for 2 h. The drying shrinkage of the S7 sample (0% sewage sludge) was 12%, while that of S1–S6 and S8–S10 samples was from 11 to 13%.

The dried samples were sintered in a laboratory furnace (Nabertherm GmbH, Lilienthal, Germany) in an oxygen atmosphere at preset temperatures (Figure 1). The time of sintering was 60 min, including 10 min at the maximum temperature. After sintering, the samples remained in the furnace until they cooled down to 100 °C.

### 2.4. Methods

The oxide composition of clay was tested with the use of an X-ray fluorescence spectrometer (XRF; PANalytical, Almeo, Netherlands). The characteristics of sewage sludge were established according to [33,34,35,36,37,38].

The phase compositions of the sewage sludge, clay, and S1–S10 samples were determined with Debye–Scherrer–Hull X-ray powder diffraction. An Empyrean diffractometer was used for this purpose (PANalytical, Almeo, The Netherlands). The test was conducted in the range of angles 6–70 °2θ with the use of a Cu lamp and an X’Celerator strip detector. The results were processed using the HighScore Plus program (v.4.7., PANalytical, Almeo, The Netherlands).

Qualitative analysis was based on the ICDD PDF-2 database. Data from the Crystallography Open Database (COD) were used for quantitative analysis, which was performed with the Rietveld method on the basis of the qualitative analysis.

Total porosity, total pore volume, and apparent density were determined with the use of a GeoPycTM 1360 density analyzer (Micromeritics, Norcross, GA, USA). The specific density of samples was determined with the use of an Acc–Pyc1330 helium pycnometer (Micromeritics, Norcross, GA, USA), and closed porosity Pz was calculated with the following formula:

(3) $$P_{z}=\left(1\;-\;\frac{d_{1}}{d_{2}}\right)100\;(\%)$$

where d_1_ is the apparent density of the sample with a preserved original structure; and d_2_ is the density of the same sample after grinding to <63 μm.

The microstructure of S1–S10 samples was examined using a Phenom XL scanning electron microscope (SEM) (Thermo Fischer Scientific, Waltham, MA, USA). The S1–S10 samples were not coated with gold for SEM. The measurements were performed with the parameters: electron beam voltage 15 kV, pressure 1 Pa. Water absorption of S1–S10 samples was determined according to [39].

## 3. Results and Discussion

**Figure 3:**
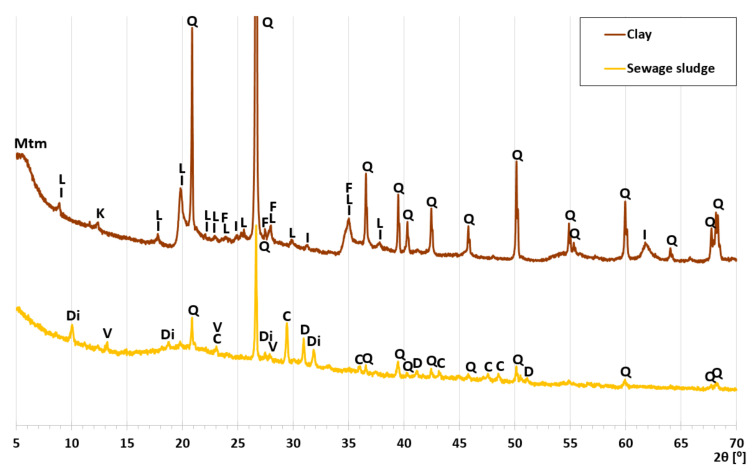
X-ray patterns of clay and sewage sludge; Q—quartz, C—calcite, D—dolomite, V—vivianite, Di—dittmarite, Mtm—montmorillonite, I—illite, K—kaolinite, F—microcline, L—muscovite, G—gypsum.

The clay contained 65.74% d.m. of silica and 15.22% d.m of alumina (Table 1). Silica and alumina are responsible for the process of growth of the liquid phase and the prolongation of an interval of mass softening during sintering [40]. The main component of the clay was quartz mixed with loamy minerals, i.e., kaolinite and illite (Figure 3, Table 2. At the beginning of the diffractogram (Figure 3), the background was higher, indicating the presence of montmorillonite. The phases identified in the clay were typical of this raw material [41].

The sewage sludge contained a considerable amount of Na_2_O and K_2_O, as well as calcium, manganese, and iron oxides (Table 3). The oxides are favorable for the formation of the liquid phase and influence its viscosity and reduce LECA expansion temperature [28,42].

Thermal expansion takes places when the mineral substance of the raw material reaches the pyroclastic state under the influence of temperature, and the released gases have sufficient pressure to increase the volume of closed pores. When the pyroclastic state is reached, the sintered raw material consists of solid, liquid, and gas phases. The solid phase is represented by crystalline forms and an amorphous phase resulting from the decomposition of loamy minerals, gypsum, and carbonates. The liquid phase is represented by eutectic solutions formed on the basis of oxides. Having sufficiently high viscosity and surface tension, the liquid phase retains the released gases in the pores. The increase of the pore size leads to an increase in the volume of LECA granules [40,43,44]. The gas phase is formed by gases resulting from the mixing water and physically bound water, as well as the products of high temperature transformations of the decomposition of carbonates, sulfates, sulphides, and organic substances and the reduction of iron oxides [30,44,45].

The sewage sludge did not contain minerals that were undesirable in the raw material for the production of LECA (Table 2). The main crystalline component was quartz, giving the most intensive peaks (Figure 3). The high background in the 15–40 °2θ range indicated a high content of the amorphous phase. Other significant components of the sewage sludge were carbonates: calcite and dolomite. Hydrated phosphate, vivianite, and dittmarite were also present in smaller amounts (Table 2). The literature shows the occurrence of vivianite [12,22,46,47] and calcite [48,49]. Therefore, calcite and dolomite from the sewage sludge may be considered the phases responsible for the release of gases, causing the expansion of samples during sintering. During the heating of vivianite and dittmarite, H_2_O and NH_3_ pairs are also released, but below 400 °C [50,51].

**Figure 4:**
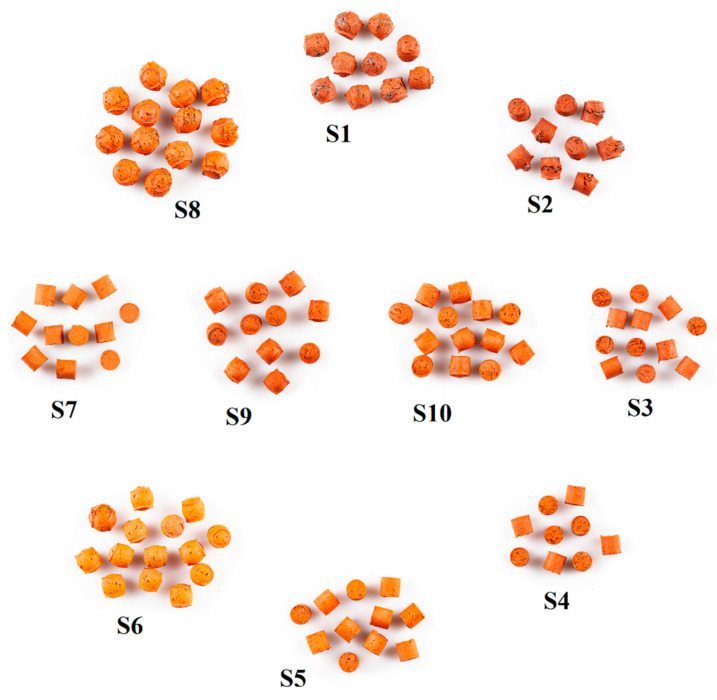
S1–S10 samples after sintering. The arrangement of samples according to the rotatable plan presented in Figure 1.

Figure 4 presents the shape of S1–S10 samples after sintering. The S6 and S8 samples were distinguished by spherical shapes, which was the effect of the expansion. The S1 and S2 samples were characterized by distinctly cracked surfaces (Figure 4). The favorable influence of the increase in the sintering temperature on the properties of LECA is consistent with the literature data [28].

**Figure 5:**
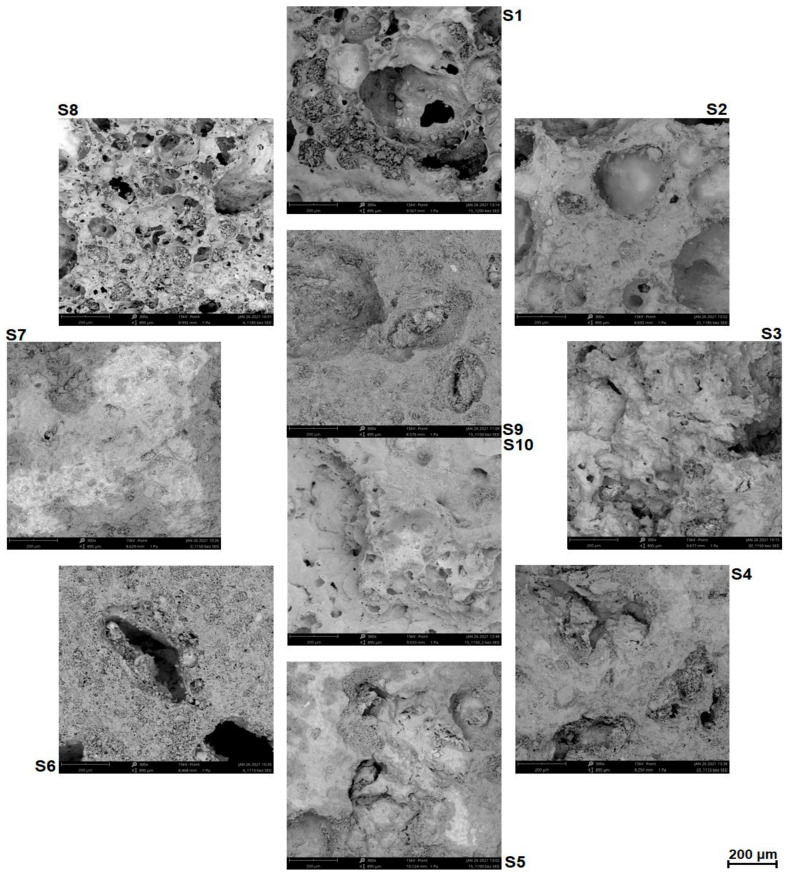
Microstructures of S1–S10 samples; magnification × 300.

The microstructure of S1–S10 samples is presented in Figure 5. The S6 and S8 samples had clear pores and cavities, similarly to the S1 and S2 samples. The presence of cavities indicated the intensive degassing of organic substance. The increase of the sewage sludge addition up to 30% changed the microstructure into a less porous one, with few, clearly smaller pores and cavities. The microstructure of the samples with 25.6% of sewage sludge was not homogenous, because in addition to big cavities (S2), less porous areas (S4) were observed. In S1, S5, S9, and S10 samples, i.e., at 15% sewage sludge content, the microstructure of LECA depended on sintering temperature. The S1 sample sintered at the highest temperature had a porous microstructure with numerous and large cavities, whereas the S5 sample sintered at the lowest temperature was less porous. Moreover, higher sintering temperature resulted in a more inhomogeneous size of pores (S1). Thus, it can be stated that the amount of the liquid phase occurring during the sintering was not sufficient to fill in empty spaces between particles. In the monitored case, the liquid phase moistened the sintered grains and facilitated their regrouping [52]. The effect of such facilitated mass transfer was the observed increased compactness of the samples.

**Figure 6:**
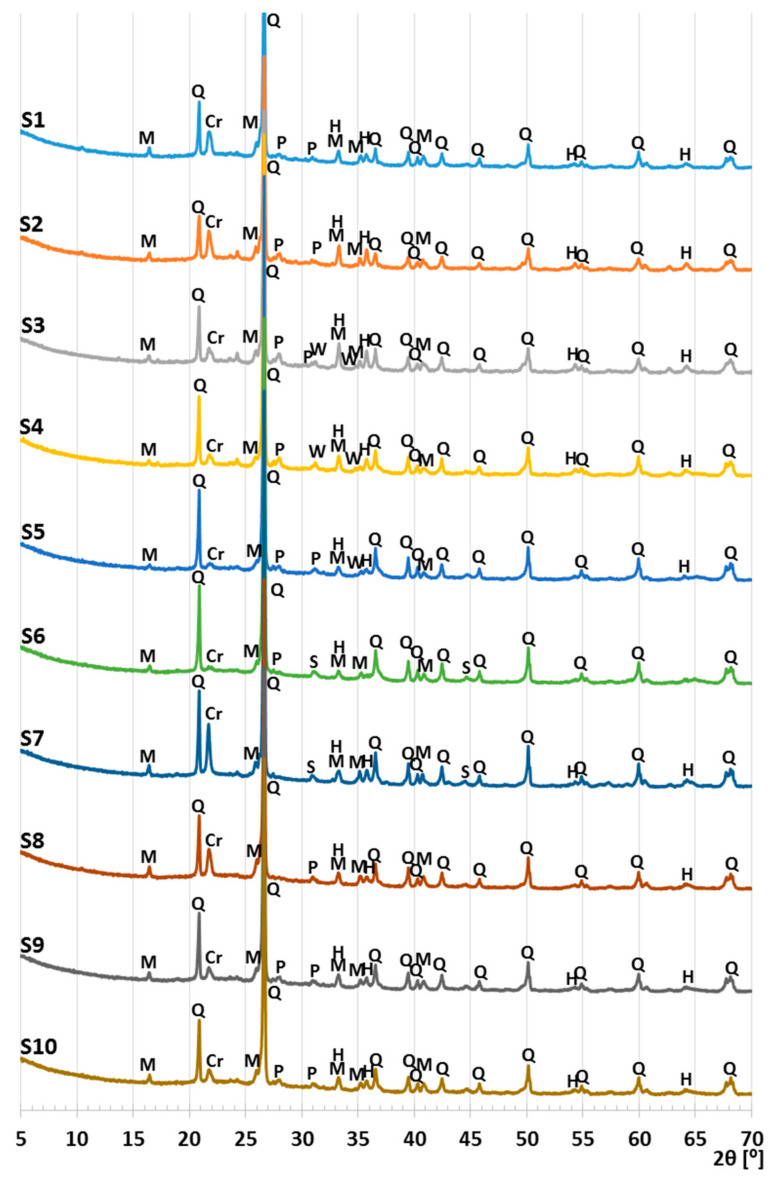
X-ray patterns of S1–S10 samples; Q—quartz, M—mullite, H—hematite, Cr—cristobalite, T—tridymite, P—plagioclase (anorthite), W—whitlockite, S—spinel.

**Figure 7:**
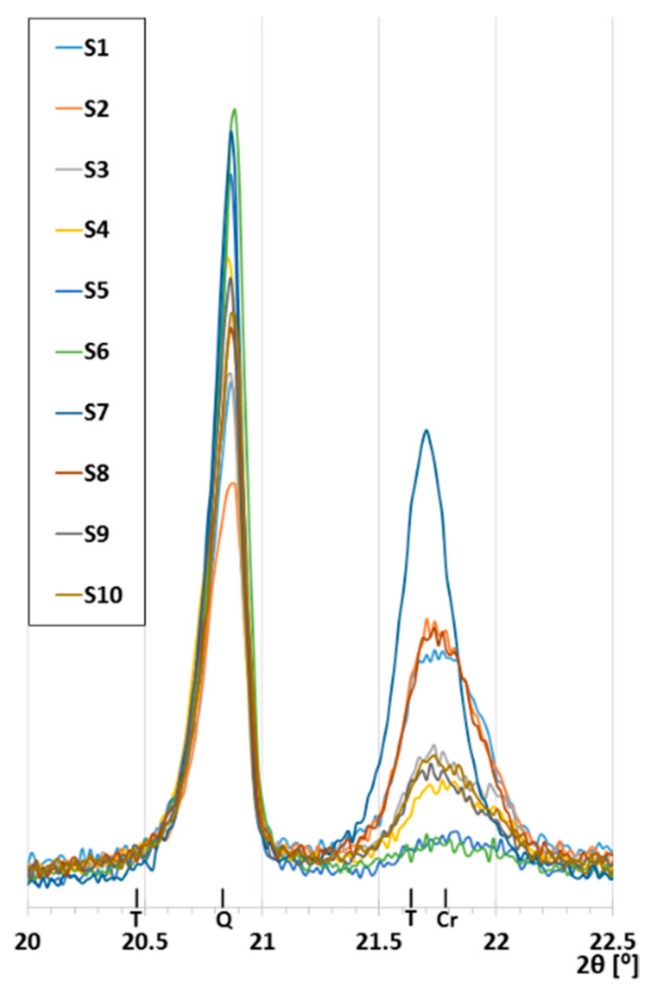
Changes of peak intensity in the ranges characteristic for quartz (Q), cristobalite (Cr), and tridymite (T).

**Figure 8:**
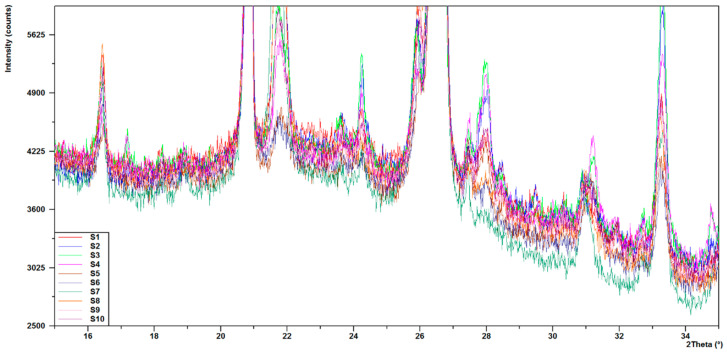
Comparison of the areas of higher background on S1–S10 diffractograms.

The comparison of the diffractograms obtained from the tests of the S1–S10 samples (Figure 6, Figure 7 and Figure 8) shows that the obtained materials were the mixtures of different forms of silica and mullite. Hematite, which influenced the color of the samples, was present in smaller quantities (Table 4, Figure 4). The samples exhibited different contents of silica, which was present in the form of quartz, metastable crystalline forms cristobalite and tridymite, as well as in the amorphous form. Significant changes were observed relative to angle 2θ = 21.7° (Figure 7). This peak consisted of the signals obtained from cristobalite and tridymite. The high background in the range 2θ 15–35° (Figure 8) indicated the presence of amorphous silica.

The highest intensity peak of cristobalite and the lowest background was present in the S7 sample, which did not contain the sewage sludge. Thus, even in the case of the formation of a glassy melt, during the lowering of the temperature, it underwent a fast crystallization, causing the formation of cristobalite.

The increase in the background level related to the presence of amorphous silica was the highest in samples S1, S2, and S8 (Figure 8). The highest background level was obtained for S1 sintered at 1200 °C. High temperature caused the meltdown of the highest amount of silica, which when cooled down, took the form of glass.

The S5 and S6 samples were characterized by very little indications for cristobalite and tridymite, and relatively low background, which were only higher than the background of the S7 sample (Figure 6, Figure 7 and Figure 8). This was the consequence of the sintering temperature (≤1115 °C). For the S4 sample, despite low sintering temperature, a clear peak from metastable crystalline silica phases was obtained. The increased content of cristobalite in S4 was related to the interaction of sewage sludge applied at a significant quantity (25.6%). Additionally, the presence of whitlockite was detected in the samples of low sintering temperature (Figure 6, Table 4).

The addition of sewage sludge to clay increased the total porosity and decreased the apparent density (Table 5). These changes were the function of the sewage sludge content. The increase in the sintering temperature was accompanied by an increase of porosity and a decrease of apparent density. The maximum value of the closed porosity (8.78424%) characterized sample S2. The relatively low values of closed porosity could result from the properties of the liquid phase at maximum temperature. In these conditions, the low viscosity of the liquid phase hindered the retention of the released gases in the samples. The fluxes from sewage sludge also had an influence on the course of the process and hence, on the physical properties of the samples (Table 2). The increase in open porosity was not accurately corelated with water absorption capability due to the glaze surfaces of the samples (Figure 4). The tested basic physical properties of samples S1–S10, i.e., specific density, apparent density, total porosity, closed porosity, sum of the pore volume, and water absorption, are important because of the diverse use of LECA. The LECA used for the production of lightweight concretes is characterized by low specific density, apparent density, and water absorption. In contrast, high values of total porosity, open porosity, and sum of pore volumes are preferred when LECA is used as a filter [14,15,16,17,18,19].

The manner in which the porous structure of LECA is formed can determine its application. LECA with high total porosity is used in the construction industry to reduce the dead weight of concrete structures, the amount of reinforcement needed in reinforced concrete buildings, and the cost of transport. High porosity materials are also used to improve thermal insulation [21].

LECA of high open porosity can be used in environmental engineering as a filtering material or in gardening [53]. The authors’ own research and literature reports confirm that heavy metals do not pose any hazard to the environment when they are immobilized in the structure of LECA [27,54].

**Figure 9:**
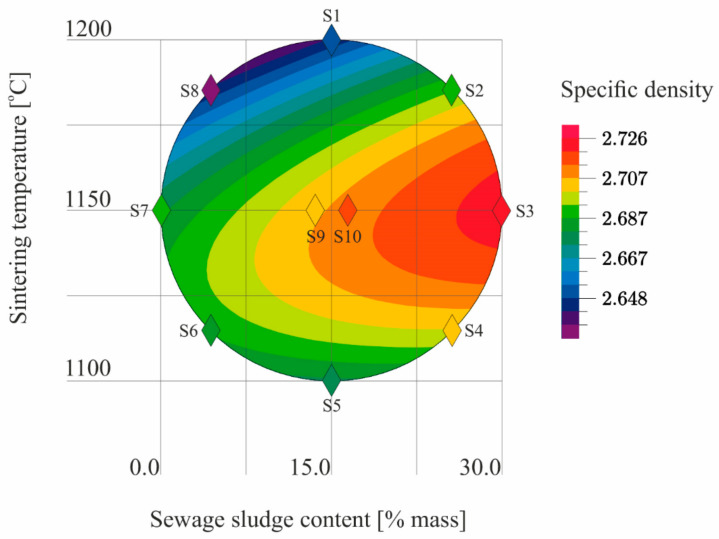
The specific density of S1–S10 samples as a function of the sewage sludge content and sintering temperature.

**Figure 10:**
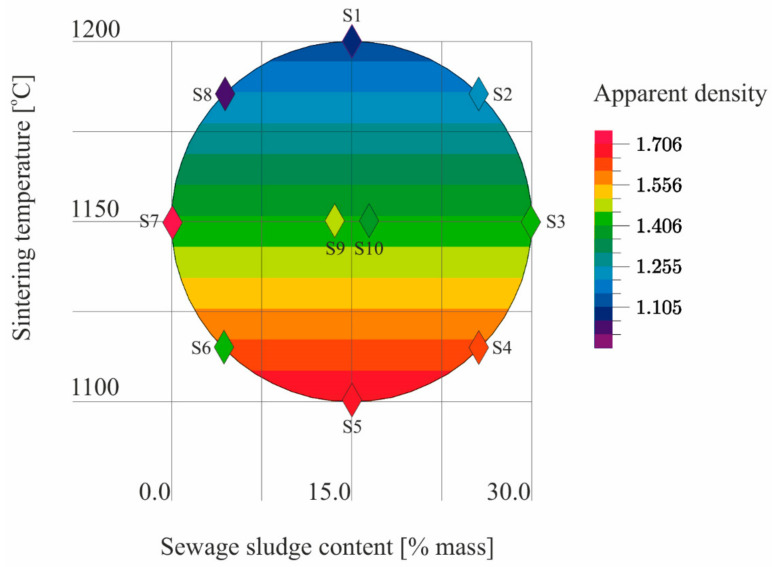
The apparent density of S1–S10 samples as a function of the sewage sludge content and the sintering temperature on the basis of the model.

**Figure 11:**
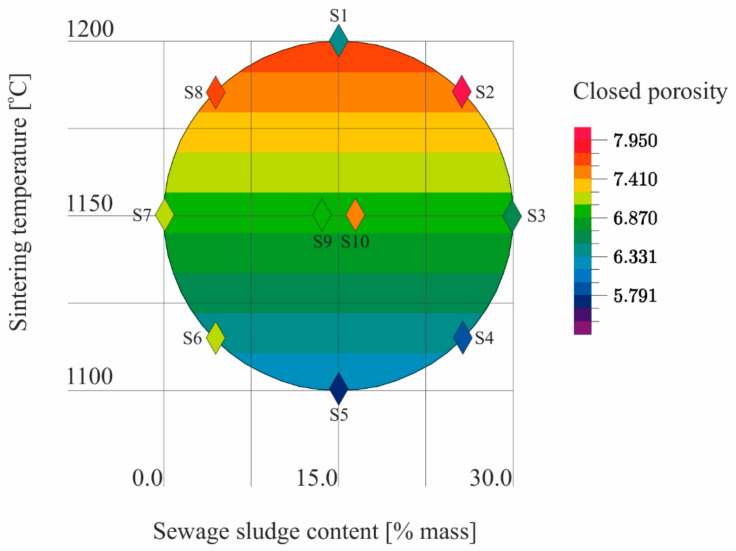
The closed porosity of S1–S10 samples as a function of the sewage sludge content and the sintering temperature on the basis of the model.

**Figure 12:**
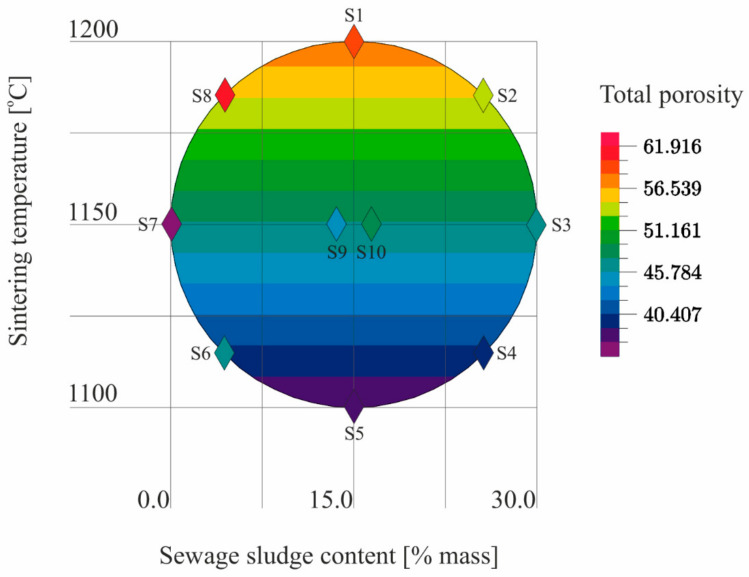
Total porosity of S1–S10 samples as a function of the sewage sludge content and the sintering temperature on the basis of the model.

**Figure 13:**
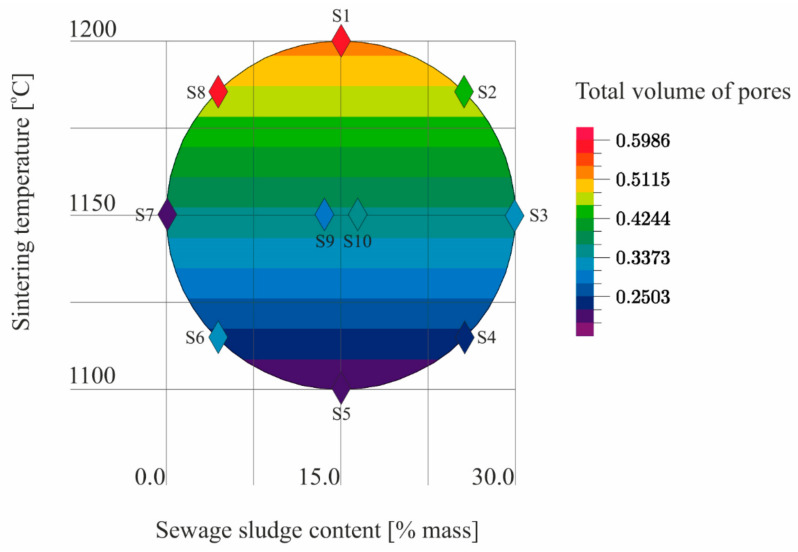
The total volume of pores of S1–S10 samples as a function of the sewage sludge content and the sintering temperature on the basis of the model.

**Figure 14:**
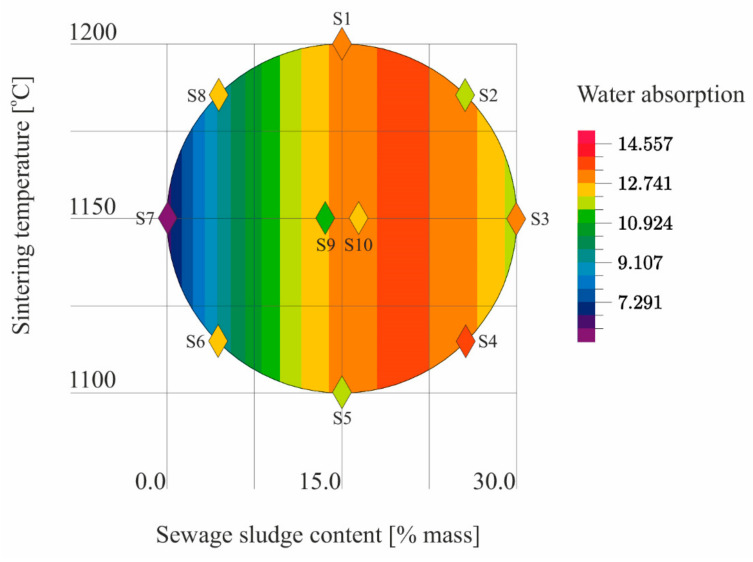
The water absorption of S1–S10 samples as a function of the sewage sludge content and the sintering temperature.

The results (Table 5) of the tests conducted according to the adopted design of the experiment (Figure 1) were the basis for the development of models describing the physical properties of the samples (Table 6). The models describe physical properties as dependent variables versus sewage sludge content (x_1_) and the sintering temperature (x_2_; Figure 9, Figure 10, Figure 11, Figure 12, Figure 13 and Figure 14).

The model describing the specific density of LECA is a quadratic relationship (Table 6). It thus follows that LECA specific density is more affected by sintering temperature changes than by the changes in the sewage sludge content. The coefficient of correlation indicated a good match between the model and empirical data. The model of the regression function confirmed that the influence of the sewage sludge content and the sintering temperature on the specific density of LECA was statistically significant (Table 6, Figure 9).

The test for the significance of the coefficient of multiple correlation (R) showed that there was a linear relationship in the regression model of apparent density. Since R = 0.8, the fit of the model to the empirical data (Table 6) was fairly good. Only the influence of sintering temperature on the LECA apparent density was statistically significant (Figure 10).

The test for the significance of R showed that there was a linear relationship in the regression model of the closed porosity. Since R = 0.6, the fit of the model to the empirical data was moderate. The obtained model showed that only the sintering temperature had a statistically significant influence on the closed porosity of LECA (Table 6, Figure 11).

There was a linear relationship in the regression model of total porosity. The R coefficient indicated a fairly good fit of the model to the empirical data (Table 6). Only the sintering temperature had a statistically significant influence on the total porosity of LECA (Figure 13). At the same time, it should be emphasized that the models of the function describing both the closed porosity and the total porosity proved a higher influence of the sintering temperature on total porosity than on closed porosity (Table 6).

There was a linear relationship in the model of the total volume of pores. The R coefficient indicated a fairly good fit to the empirical data. The influence of sintering temperature on the total volume of pores was statistically significant (Table 6, Figure 13).

The model was obtained after testing the significance of regression coefficients describing water absorption of the samples at the significance level *p* = 0.2. The model (Table 6) is a quadratic relationship, indicating that the effect of sintering temperature on the water absorption of LECA is stronger than that of sludge content. The R coefficient indicated a moderate fit of the model to the empirical data (Table 6, Figure 14).

Table 7 presents the results of the Pearson correlation analysis for the properties of LECA. The obtained results indicated the presence of strong correlations between the bulk density, the total porosity, and the total volume of pores, as well as between the total porosity and the total volume of pores. However, no major correlation existed between the water absorption and the closed porosity as well as the water absorption, the total porosity and the total volume of pores.

## 4. Conclusions

The test results demonstrated that the sewage sludge content influences the water absorption and specific density of LECA. The sintering temperature has an effect on the specific density, apparent density, total porosity, closed porosity, total pore volume, and water absorption of LECA. There is no correlation between closed porosity and water absorption of the tested samples. This was due to the formation of the amorphous phase during the sintering of samples. The formation of the amorphous phase was promoted by the fluxes from the clay and sewage sludge. Higher sewage sludge content was responsible for a clear influence of fluxes on the microstructure of LECA.

The study confirmed that sewage sludge can be added to the raw material mass for the production of LECA, which is beneficial because it increases the tolerance of the technological formula to variations of the raw material composition.

Striving for the maximum content of sewage sludge with the simultaneous preservation of the desired properties of LECA is consistent with sustainable development principles. The use of sewage sludge in the production of LECA unquestionably reduces the use of natural raw materials and solves the problem of its management. Moreover, this method of utilization of sewage sludge is a form of the recovery of energy embodied in the biomass of sewage sludge, which fits the paradigm—the model of economy based on the utilization of renewable sources of energy.

## Figures and Tables

**Table 1:** Oxide composition of clay, % d.m.

Oxide	SiO_2_	Al_2_O_3_	Fe_2_O_3_	Na_2_O	K_2_O	MgO	TiO_2_	CaO	Mn_2_O_3_	SO_3_	BaO	P_2_O_5_	Cr_2_O_3_
Clay	65.74	15.22	4.54	0.17	1.75	1.18	0.85	0.70	0.02	0.01	0.019	0.04	0.02

**Table 2:** Crystalline phases in clay and sewage sludge, % d.m.

Component	Q	C	D	V	Di	L	K	I	F	Mtm	G
Clay	37.9	-	-	-	-	21.4	20.9	11.2	7.5	0.3	0.8
Sewage Sludge	37.0	25.2	22.3	9.8	5.7		-	-	-	-	-

Q—quartz, C—calcite, D—dolomite, V—vivianite, Di—dittmarite, Mtm—montmorillonite, I—illite, K—kaolinite, F—microcline, L—muscovite, G—gypsum.

**Table 3:** Characteristics of sewage sludge.

pH	Water Content %	Dry Mass, % d.m.	Organic Substances, % d.m.	Mineral Substances, % d.m.		P_general_ % d.m.		N_general_ % d.m.		Ca mg·kg^−1^ d.m.
8.20	80.4	19.6	58.3	41.7		2.4		3.1		3065.0
Metals, mg·kg^−1^ d.m.										
Pb	Cd	Hg	Cr	Cu	Zn	Fe	Mg	Mn	Na	K
16.7	1.0	0.03	61.6	9.1	838.0	1073.3	1849.1	102.3	212.8	750.9

**Table 4:** Phase composition of S1–S10 samples, %.

Components	S1 *	S2 *	S3 *	S4 *	S5 *	S6 *	S7 *	S8 *	S9 *	S10 *
Quartz	51.4	52.0	59.4	67.0	73.0	64.1	58.0	53.2	64.9	64.3
Mullite	30.7	25.2	22.1	20.9	19.9	20.4	23.8	32.1	27.4	27.6
Hematite	2.0	6.8	8.9	3.3	1.4	0.7	1.4	1.1	2.1	2.2
Cristobalite	13.4	12.5	2.7	2.2	1.3	0.9	13.1	11.5	2.3	2.5
Tridymite	1.9	1.3	2.2	1.9	1.8	1.8	1.4	1.7	2.3	2.5
Plagioclase	0.8	1.2	1.3	1.3	0.9	0.6	-	0.4	1.0	1.0
Whitlockite	-	-	3.4	3.4	1.7	-	-	-	-	-
Spinel	-	-	-	-	-	11.5	2.3	-	-	-

* The test sample was made by grinding of the five granules.

**Table 5:** Physical properties of S1–S10 samples.

Sample	Specific Density, g·cm^−3^	Apparent Density, g·cm^−3^	Closed Porosity, %	Total Porosity, %	Total Volume of Pores, cm^3^·g^−1^	Water Absorption, %
S1	2.65256 ± 0.01100	1.06784 ± 0.10675	6.35394 ± 1.16434	59.7508 ± 3.9428	0.56726 ± 0.09742	13.22 ± 1.12
S2	2.68818 ± 0.01912	1.24884 ± 0.04168	8.78424 ± 0.78057	53.5416 ± 1.5176	0.42934 ± 0.02589	11.68 ± 0.69
S3	2.72156 ± 0.006946	1.45206 ± 0.13281	6.65904 ± 0.47699	46.7108 ± 4.9416	0.32572 ± 0.06208	12.88 ± 0.86
S4	2.70182 ± 0.00335	1.64276 ± 0.01107	5.8717 ± 0.34249	39.1964 ± 0.4019	0.23856 ± 0.00405	13.60 ± 1.50
S5	2.67886 ± 0.01394	1.67188 ± 0.02028	5.72246 ± 0.38199	37.5898 ± 0.5501	0.22468 ± 0.00607	12.02 ± 0.48
S6	2.68284 ± 0.01404	1.42768 ± 0.03774	7.00168 ± 0.33496	46.7808 ± 1.4701	0.32802 ± 0.01892	12.50 ± 1.04
S7	2.68918 ± 0.02028	1.74602 ± 0.27044	7.07420 ± 1.06224	35.0640 ± 10.1908	0.21026 ± 0.07740	2.20 ± 0.71
S8	2.63116 ± 0.00449	1.02612 ± 0.03303	7.61958 ± 0.21512	60.9998 ± 1.2469	0.5952 ± 0.03194	12.68 ± 1.17
S9	2.70062 ± 0.00993	1.46924 ± 0.02088	6.93252 ± 0.27298	45.5948 ± 0.8041	0.3104 ± 0.00991	11.20 ± 1.88
S10	2.71372 ± 0.00800	1.39032 ± 0.14738	7.43658 ± 0.78397	48.7664 ± 5.4594	0.35812 ± 0.08802	12.24 ± 1.34

**Table 6:** Models describing the tested physical properties of LECA.

Dependent Variable (y_i_)	Regression Model	Coefficient of Multiple Correlation
specific density	y = 2.70341 + 0.02153·x_1_ − 0.01812·x_2_ − 0.04341·x_2_^2^	R = 0.9
apparent density	y = 1.41428 − 0.29165·x_2_	R = 0.8
closed porosity	y = 6.94559 + 07.8199·x_2_	R = 0.6
total porosity	y = 47.39952 + 10.59057·x_2_	R = 0.8
total volume of pores	y = 0.35876 + 0.16662·x_2_	R = 0.8
water absorption	y = 12.95868 + 2.68808·x_1_ − 3.84228·x_2_^2^	R = 0.7

x_1_—sewage sludge content % d.m, x_2_—sintering temperature °C.

**Table 7:** Pearson’s correlation coefficients between physical properties of samples S1–S10.

Physical Properties	Specific Density	Apparent Density	Closed Porosity	Total Porosity	Total Volume of Pores	Water Absorption
Specific density	1.0000					
Apparent density	0.6162	1.0000				
Closed porosity	−0.0819	−0.4612	1.0000			
Total porosity	−0.5811	−**0.9990**	0.4699	1.0000		
Total volume of pores	−0.6957	−**0.9885**	0.4138	**0.9832**	1.0000	
Water absorption	−0.0734	−0.4855	−0.1482	0.4978	0.4096	1.0000

Bold—significant correlations at *p* < 0.05.

## Data Availability

Not applicable.
